# From direct scoring to cue integration: big five prediction from spoken responses to a picture-based self-projection task

**DOI:** 10.3389/fpsyg.2026.1930314

**Published:** 2026-09-30

**Authors:** Qifan Yang, Jian Li

**Affiliations:** Faculty of Psychology, Beijing Normal University, Beijing, China

**Keywords:** big five personality, cue integration, LLM-assisted measurement, picture-based self-projection task, spoken responses, trait recoverability

## Abstract

**Objective:**

Open-ended language tasks are increasingly used in personality assessment, but their psychometric value largely depends on two factors: the type of evidence a task elicits and how that evidence is represented before scoring.

**Methods:**

In this study, we examined five spoken responses to a picture-based self-projection task collected from 374 participants during in-person workplace training sessions. Participants first completed the Big Five Inventory-2 questionnaire; the spoken task was administered approximately 1 day later, and responses were transcribed using a Whisper-based automatic speech recognition pipeline. We compared four approaches applied to the same transcribed corpus: Linguistic Inquiry and Word Count (a lexical dictionary method), contextual semantic embeddings, holistic large language model (LLM) appraisal (holistic appraisal route; HAR), and hierarchical cue integration route (HCIR).

**Results:**

The results were trait-specific: extraversion was the most recoverable domain across methods, whereas Openness was the least recoverable. HAR preserved rank-order information, particularly for Extraversion, but exhibited poor calibration, evidenced by negative mean *R*² values and systematic directional bias in extreme score bands. In the retained comparison, HCIR-Grounded showed the most favorable observed association–calibration profile among the evaluated approaches (mean |*r*| = 0.264, mean *R*^2^ = 0.073), with notably higher observed correlations for Agreeableness, Conscientiousness, and Neuroticism.

**Discussion:**

We refrain from attributing this pattern solely to cue integration, however, as HCIR included a supervised calibration stage that HAR lacked; the comparison therefore reflects the joint contribution of cue organization and supervised calibration. By explicitly organizing cue provenance, self-relevance, quality indicators, and trait-specific evidence before supervised calibration, HCIR supports a measurement-oriented interpretation of LLM-assisted personality inference from spoken responses that combine self-projection, preference, and normative evaluation.

## Graphical abstract

**Figure:**
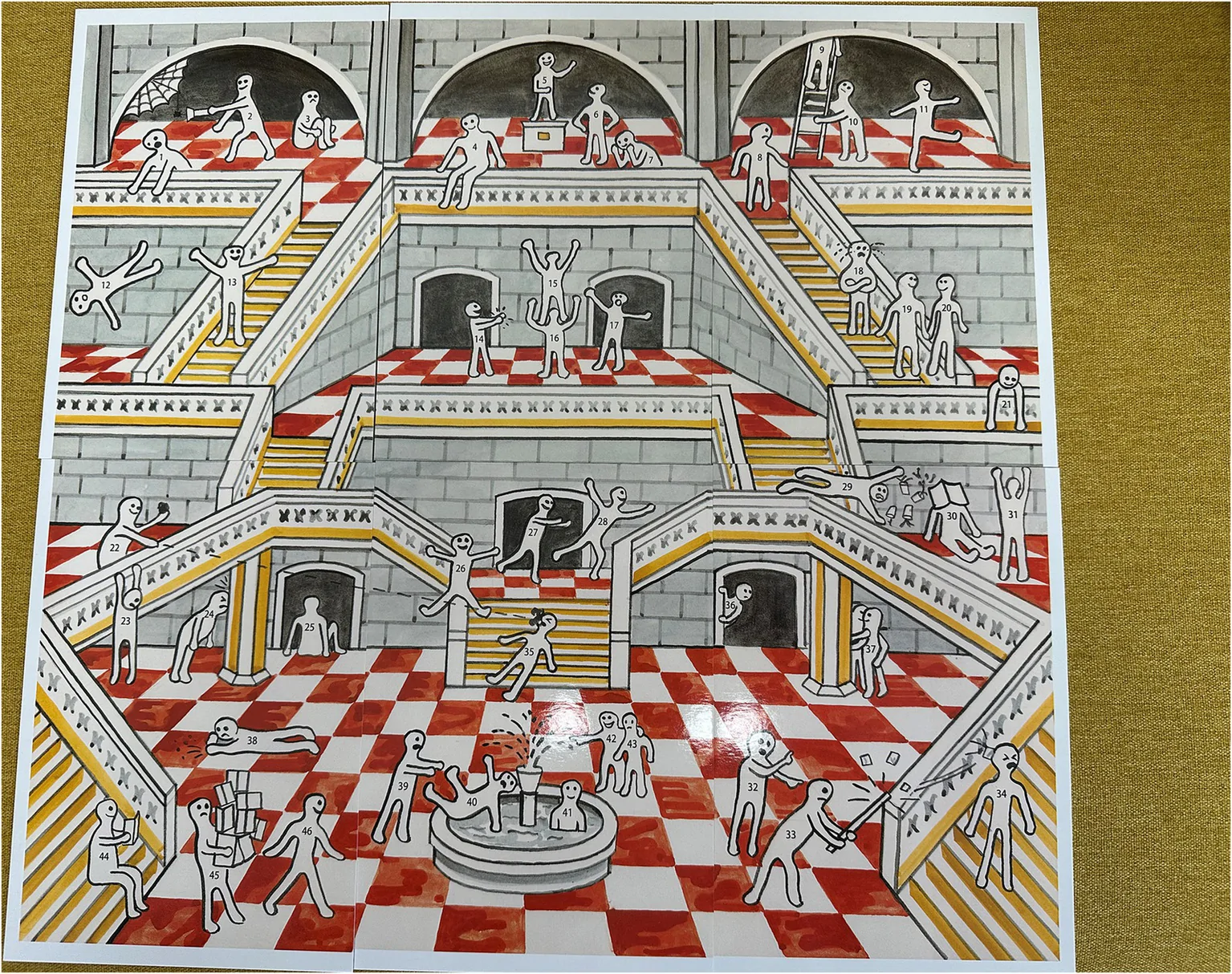
Stimulus Figure: Picture-based self-projection stimulus used to elicit the five open-ended spoken responses. The full stimulus scene is shown without participant-specific annotations. Participants selected characters from the shared scene and explained their choices in work, family, friends, and preferred- and disliked-character prompts. The term “self-projection” describes the task format and does not imply a traditional clinical projective test.

## 1 Introduction

Language-based personality assessment has evolved from lexical dictionaries to contextual embeddings and, most recently, large language models (LLMs). However, this expansion has not resolved the fundamental measurement problem, instead bringing that problem into sharper focus. The Big Five personality provides a well-established structure for describing broad personality domains (McCrae and Costa, 1987; Goldberg, 1990; McCrae and Costa, 1997), and language has repeatedly been shown to contain personality-relevant information in blogs, conversations, essays, social media posts, and other digital traces (Pennebaker and King, 1999; Mairesse et al., 2007; Fast and Funder, 2008; Yarkoni, 2010; Park et al., 2015). Contemporary reviews and LLM-based studies extend this work, but they also underscore that text-based inference depends critically on the source text, validation criterion, trait observability, and representation used before prediction (Boyd and Pennebaker, 2017; Boyd and Schwartz, 2021; Eichstaedt et al., 2021; Koutsoumpis et al., 2022; Hinds and Joinson, 2024; Peters and Matz, 2024; Wright et al., 2026).

These developments are best understood as shifts in the unit of representation. Dictionary methods count psychologically interpretable word categories, embedding methods represent utterances in dense semantic spaces, and LLM-based methods can summarize, reason over, and score open-ended responses. Each step increases representational flexibility, but flexibility alone does not guarantee measurement validity. A model might detect semantic regularities that covary with personality labels without preserving the reasons for those regularities being relevant. Conversely, a transparent method might be psychologically interpretable but insufficiently fine to recover the cue structure present in a short response. The present comparison treats these approaches as different answers to the same measurement question: what information is retained between the raw spoken response and the final trait score?

Therefore, the key issue is not simply whether text can predict personality, but rather what type of evidence a task elicits, how that evidence is represented, whether model outputs are calibrated to a questionnaire scale, and whether recoverability differs systematically across Big Five domains. A scoring procedure might preserve rank-order association while distorting scale dispersion; it might reduce average error by shrinking predictions toward the sample center. In asymmetric trait distributions, such shrinkage is not neutral: it can specifically suppress the score regions that carry important information on individual differences. Consequently, correlation, calibration, error magnitude, and signed error address fundamentally different measurement questions.

This study used a brief picture-based spoken response task in which participants viewed a shared scene with numbered characters and responded aloud to five prompts: three self-projection prompts identifying the character most like themselves in work, family, and friendship contexts, and two evaluation prompts identifying the characters they liked most and disliked most. The term “projective” is used descriptively to refer to picture-based self-projection and character evaluation, not to traditional clinical projective tests. Unlike autobiographical essays, diaries, social media posts, and unconstrained conversation, where cues could accumulate across many occasions (Mehl et al., 2006; Schwartz et al., 2013; Stachl et al., 2020), this task captures a constrained set of responses. Unlike forced-choice and Likert items, respondents decide which aspects of the scene to attend to, which character to select, and which reasons to articulate. The resulting evidence is therefore partly self-descriptive, partly interpretive, and partly evaluative.

The five prompts that we used were not psychologically equivalent: the three self-projection prompts were closer to grounded self-description, whereas the preferred- and disliked-character prompts more readily invited aspiration, value preference, rejection criteria, moral judgment, and normative evaluation. Through a Brunswikian lens, these prompts sample different cue ecologies (Brunswik, 1956). In the realistic accuracy model of personality judgment, cue validity and cue use vary systematically with observability, evaluative load, and self-relevance (Funder, 1995; Letzring et al., 2006; Biesanz et al., 2007; Vazire, 2010; Funder, 2012). Trait activation theory offers a complementary vocabulary, in which prompts differ in the trait-relevant situations they create and the dispositions they make easier, or harder, to express (Tett and Guterman, 2000; Tett and Burnett, 2003). Together, these perspectives converge on a single prediction: prompt heterogeneity should shape Big Five recoverability.

This prompt heterogeneity has direct implications for Big Five recoverability. Extraversion should be relatively visible when a task requires respondents to position themselves in social scenes and explain interpersonal choices. Agreeableness and Conscientiousness might require disentangling prosocial stance, duty-related content, and self-regulatory behavior from socially desirable self-presentation; neuroticism might be suppressed in a workplace training context where distress, vulnerability, and instability are less acceptable to disclose; and openness could be constrained when the task limits imaginative range and intellectual exploration. Therefore, it should not be assumed that the same response set diagnoses all five domains equally effectively.

The contrast between direct scoring and cue integration is a consequence of this heterogeneity. Direct LLM scoring treats the response set as sufficient input for a final trait judgment. This approach can recover useful rank-order information, but it can also compress heterogeneous evidence into a global impression. For example, a respondent might sound warm when describing a liked character, dutiful when describing an ideal work role, and critical when rejecting a disliked character. A direct score has to implicitly decide how much of that material reflects the respondent’s stable trait standing rather than preference, aspiration, or normative judgment. A cue-integrated approach makes a different measurement commitment: it first preserves prompt function, source type, self-relevance, cue quality, and trait-specific content, and then calibrates those cues against questionnaire labels. From the perspective of construct validation, this approach makes the evidentiary basis of the score more inspectable before final prediction, a feature that is largely absent in end-to-end direct scoring (Cronbach and Meehl, 1955).

This study had three aims: to test whether five spoken responses to a picture-based self-projection task contained usable signal for Big Five prediction; to examine whether recoverability differed across the five domains; and to compare lexical, semantic-embedding, direct LLM scoring, and cue-integrated approaches in terms of association, calibration, and evidentiary inspectability. These aims were intentionally descriptive rather than framed as confirmatory hypotheses about a single winning model. The goal was to evaluate how different representations behave when the same heterogeneous spoken evidence is mapped onto the same questionnaire criterion, and to identify which failures are better understood as task evidence, representation, or calibration limits.

This study contributes to the literature in three ways: it provides a systematic comparison of four approaches on a held-constant spoken-response corpus, directly quantifying the trade-offs between rank-order association and scale calibration; it introduces and evaluates a hierarchical cue integration framework that preserves evidence provenance (an intermediate layer that makes the basis of LLM-assisted personality inference more inspectable than end-to-end scoring); and, by documenting systematic directional bias in direct LLM scoring, including overprediction of low Agreeableness and low Conscientiousness and underprediction of high Neuroticism and high Openness, it highlights a major calibration problem that pure correlation analysis fails to reveal. This logical framework also guides the presentation of both valid predictive patterns and insufficient trait prediction results.

## 2 Method

### 2.1 Participants, setting, and data collection

Data were collected on an in-person basis during workplace training sessions conducted offline. Participants were recruited voluntarily, and no compensation was provided beyond the training content itself. The analytic sample consisted of 374 participants with questionnaire records that could be reliably aligned with corresponding voice response records. All retained cases were stored under anonymized participant identifiers (PIDs), and alignment was performed according to PID. Data cleaning was primarily based on record linkage and the usability of voice response materials for transcription and subsequent modeling. There were no additional exclusion criteria beyond record linkage failures and unusable voice recordings (e.g., empty files, severe truncation).

Participants were aged 18–56 years (mean = 23.94 ± 2.54 years; median = 24 years). Gender was available for all participants and, in the archived scoring file, was coded as 1 = male and 2 = female. According to this coding, 146 participants (39.0%) were male, and 228 (61.0%) were female. The sample consisted predominantly of working adults (*n* = 334, 89.3%), with a smaller subgroup of university students (*n* = 40, 10.7%). Educational attainment was mainly at the bachelor’s (*n* = 189, 50.5%) and master’s (*n* = 185, 49.5%) levels. The participant characteristics are summarized in Table 1.

**Table 1:** Descriptive characteristics of the workplace training sample.

Characteristic	Statistic
Participants	374
Age (years)	M = 23.94, SD = 2.54, Median = 24, Range = 18–56
Male	146 (39.0%)
Female	228 (61.0%)
Identity group: Working adults	334 (89.3%)
Identity group: University students	40 (10.7%)
Education: Bachelor’s degree	189 (50.5%)
Education: Master’s degree	185 (49.5%)

Percentages are based on the analytic sample with valid PID linkage (*N* = 374). Gender was coded in the archived scoring file as 1 = male and 2 = female.

The study was conducted in accordance with applicable ethical standards, and all participants provided informed consent before taking part.

The workplace training setting might have increased the ecological relevance of work-related self-projection while simultaneously encouraging norm-consistent self-presentation. The participants responded in a context where role adaptation, discipline, cooperation, and entry into organizational life were likely salient. This context should be considered when interpreting both the strength of the work-related cues and the compression of socially undesirable trait expressions (Ones et al., 1996; Birkeland et al., 2006; Morgeson et al., 2007).

### 2.2 Task order and criterion measure

The participants first completed the Big Five Inventory-2 (BFI-2) questionnaire. The picture-based spoken response task was administered approximately 1 day later as a separate mobile module. Therefore, the two assessments were separated by approximately 24 h, although the order was fixed.

The criterion measure was the score on the 60-item BFI-2 (Soto and John, 2017). The BFI-2 assesses Extraversion, Agreeableness, Conscientiousness, Negative Emotionality, and Open-Mindedness, with three facets per domain. The Chinese version of the BFI-2-60 has been evaluated in diverse Chinese samples, with evidence of internal consistency, hierarchical domain-facet structure, convergent and discriminant validity, and criterion-related validity (Zhang et al., 2022). The 60-item version was appropriate for our analysis because a brief spoken task might capture certain facets within a broad domain more clearly than other tasks; therefore, a criterion measure with broad domain coverage is preferable to an extremely brief personality screener. The analyses used the five broad-domain scores as criterion variables. Negative Emotionality was discussed as Neuroticism and Open-Mindedness as Openness for consistency with the rest of the manuscript. The internal consistency of the BFI-2 domain scores in the present sample was acceptable, with Cronbach’s *α* values of 0.82–0.89 across the five domains (Extraversion, 0.85; Agreeableness, 0.83; Conscientiousness, 0.88; Neuroticism, 0.82; Openness, 0.87).

After recoding reverse-scored items, domain means were computed on the original 1–5 BFI-2 response metric. For consistency with the modeling workflow, each domain mean was linearly transformed to a 1–9 scale using the following formula: transformed score = 2 × original mean − 1. Therefore, original scores of 1, 3, and 5 corresponded to transformed scores of 1, 5, and 9, respectively. The choice of a 9-point output scale for HAR, rather than directly matching the 1–5 BFI-2 metric, was methodological. We opted for a wider response range to allow LLMs to express graded trait judgments with sufficient granularity, based on pilot observations that LLM-generated scores on a 1–5 scale tended to concentrate on the midpoints (3 and 4), reducing discriminative variance. The 1–9 format offered additional response categories in the hope of mitigating this compression and preserving more individual differences. However, this design choice introduced a measurement invariance concern: linear rescaling from 1–9 to 1–5 assumes that scale intervals are equivalent across the two metrics and that LLMs use the 1–9 scale in a manner comparable to human questionnaire respondents. This assumption is unlikely to hold perfectly. Cross-scale transformations may introduce systematic biases if LLMs systematically avoid scale endpoints or treat middle categories differently from human participants. Therefore, the calibration failure observed for HAR should be interpreted with this rescaling decision in mind. Future work should directly prompt LLMs on the target 1–5 metric or conduct equivalence testing to establish measurement invariance before comparing LLM-generated scores with questionnaire criteria.

All five criterion dimensions covered the observed range, but the score distributions were asymmetric: Agreeableness, Conscientiousness, and Openness were shifted upward, whereas Neuroticism was comparatively low. Table 2 reports the means, SDs, quartiles, skewnesses, and proportions of participants in the low- and high-score bands.

**Table 2:** Distributional characteristics of the BFI-2 domain labels in the analytic sample.

Trait	N	Mean	SD	P25	Median	P75	Skewness	Low (1–3) %	High (7–9) %
Extraversion	374	5.595	2.005	4	6	7	−0.215	16.6%	34.2%
Agreeableness	374	6.135	1.986	5	6	8	−0.252	10.2%	39.8%
Conscientiousness	374	6.064	1.870	5	6	7	−0.542	9.4%	43.3%
Neuroticism	374	3.707	1.765	3	4	5	0.560	47.3%	7.0%
Openness	374	5.909	1.914	5	6	7	−0.397	10.7%	39.0%

Domain labels were stored on the 1–9 scale used throughout the modeling workflow. P25 and P75 denote the 25th and 75th percentiles, respectively. Negative skew indicates concentration at higher scores, whereas positive skew indicates concentration at lower scores.

The distributional pattern affected how performance should be interpreted. A model can reduce mean absolute error (MAE) or root mean square error (RMSE) by drifting toward the sample center or toward the more common side of the scale. In asymmetric label distributions, this behavior is a measurement issue rather than a purely technical smoothing effect. Therefore, the analyses considered rank-order association, *R*^2^, error magnitude, and signed error together.

### 2.3 Materials, prompts, response procedure, and transcription

The task used a shared picture stimulus populated by numbered characters. During the mobile module, participants used their own smartphones and earphones to view the stimulus on the Credamo platform (a Chinese online data collection platform) and record spoken answers to five prompts. Recording was accomplished in self-selected quiet environments using personal devices; no specific acoustic constraints were imposed. The self-projection prompt set included work, family, and friends, followed by preferred- and disliked-character prompts. Participants identified the character most similar to themselves in work, family, and friendship contexts and explained why. They then identified the characters they liked most and liked least and explained their choices.

The first three prompts were treated as self-projection prompts because they asked participants to locate themselves in everyday social contexts. The final two prompts were treated as preference and evaluation prompts because they asked participants to express attraction, rejection, and judgment toward the characters in the scene. These prompt function differences were central to the method comparison: direct scoring could collapse them into a single impression, whereas cue integration was designed to preserve their provenance. The work prompt was expected to make responsibility, execution, role adaptation, and pressure management more salient, whereas the family and friendship prompts were expected to make affiliation, dependence, support, boundaries, and participation more visible. The preferred-character prompt could contain self-idealization or valued traits, whereas the disliked-character prompt could reveal rejection criteria and moral boundaries without necessarily describing the respondent’s own behavior.

Audio responses were transcribed using a Whisper-based automatic speech recognition pipeline (i.e., the “large-v3” model; Radford et al. (2023)). Before modeling, transcripts were screened for linkage consistency, empty outputs, severe truncation, prompt mismatch, and obvious non-speech artifacts. To further assess transcription quality, approximately 10% of valid participant records (*n* = 37) were compared with manually transcribed reference texts. The character error rate (CER), computed as the sum of substitution, deletion, and insertion errors divided by the number of characters in the reference transcript, varied across participants (overall CER = 0.08 [8%]). This audit was used to characterize transcript quality before downstream text-based modeling.

Transcript quality was considered acceptable for downstream aggregate-level text modeling, although transcription noise remained possible. The present comparison used transcripts only and did not include acoustic or prosodic information such as pauses, speech rate, intensity, or vocal affect, even though such cues can carry affective and personality-relevant information (Banse and Scherer, 1996; Scherer, 2003; Low et al., 2020).

Prompt-level descriptive summaries, including response length, missingness, self-relevance indicators, and inferred prompt function, are reported in Table 3. Because the same transcript corpus was used in all approaches, differences among approaches reflect evidence representation and scoring strategy rather than differences in the underlying responses.

**Table 3:** Prompt-level characteristics of the five picture-based spoken responses, including the response length, missingness, self-relevance, and inferred function.

Prompt	Length M	SD	Missing (%)	Direct-self (%)	Idealized/low-self (%)	Dominant source	Primary function	Primary risk
Work self-projection	180.18	86.63	0.0%	82.4%	9.6%	Self-behavior	Task orientation, stress tolerance, execution chain	May invite idealized work selves or performative maturity.
Family self-projection	156.90	67.84	0.0%	86.4%	5.1%	Self-behavior	Dependency, care, everyday preference, stability needs	May reflect comfort preference rather than stable personality.
Friends self-projection	144.10	56.12	0.0%	91.4%	1.6%	Self-behavior	Social participation, boundaries, interaction style	Lively scenes may be overread as Extraversion.
Preferred character	149.42	59.60	0.0%	3.5%	75.7%	Self-value	Aspirational traits, value preference, idealized self	Idealized projection and social desirability are strong.
Disliked character	148.75	71.62	4.8%	0.3%	85.6%	Moral norm	Rejection criteria, moral boundaries, risk sensitivity	Normative condemnation may replace self-description.

Length M denotes the mean response length in Chinese characters. Direct-self % and Idealized/low-self % values were derived from the archived Stage 1 prompt-based pipeline rather than from an independent human-coding round. Dominant source, Primary function, and Primary risk summarize the task-design logic rather than external ground truth.

### 2.4 Scoring approaches

All four approaches operated on the same participant-level transcript corpus. “Approach” refers to the broad model comparison category, and “route” is retained in the names of the two LLM-based scoring architectures, HAR and HCIR. An overall workflow diagram summarizing the LLM-based pipelines is provided in Supplementary Figure S1.

Linguistic Inquiry and Word Count (LIWC). This lexical baseline used LIWC-derived features with RidgeCV (ridge regression with cross-validation) to test whether psychologically interpretable surface categories alone could recover trait variance (Tausczik and Pennebaker, 2010; Pennebaker et al., 2015). We used the LIWC2015 dictionary with 93 standard categories. We did not expect LIWC to capture all semantic content, but it provided an interpretable low-complexity comparison, and its value was methodological: if LIWC performed well, more complex models would need to justify their opacity, whereas weak performance would indicate that surface lexical categories inadequately represented the relevant evidence.

Contextual semantic embeddings (CSEM). This contextual semantic baseline used Qwen3 embeddings to represent the same text. During early development, we also explored BGE-M3 as an alternative embedding backbone; however, Qwen3 consistently outperformed BGE-M3 in preliminary validation and was therefore selected as the primary embedding model for the integrated comparison. We used Qwen3-7B-Instruct (Hugging Face model ID: Qwen/Qwen3-7B-Instruct; hidden dimension = 3,584) to extract sentence-level embeddings from each transcribed response. For each participant, the five prompt responses were embedded independently by mean-pooling the last hidden layer across token positions.

We compared two embedding aggregation strategies. The global-only variant used the mean of the five prompt embeddings as the single text representation for each participant. The global-plus-difference variant additionally computed, for each of the five prompts, the difference between that prompt’s embedding and the participant’s global mean embedding. These five difference vectors were then concatenated with the global vector to form the final feature representation (total dimension = 3,584 × 6 = 21,504). This design was intended to preserve both the participant’s overall semantic baseline and prompt-specific deviations from that baseline. The global-plus-difference variant outperformed the global-only variant in preliminary validation and was therefore advanced to the integrated comparison.

CSEM tested whether modern embeddings improve prediction beyond lexical categories, while leaving prompt function, source type, and cue quality implicit. After feature construction, the high-dimensional feature matrix was reduced via principal component analysis (PCA), retaining components that explained 95% of the variance (approximately 120 components). The original participant-level out-of-fold predictions were reconstructed from the archived PCA-reduced feature matrices and the original fivefold RidgeCV workflow, and are labeled as “reconstructed” throughout the manuscript.

### 2.5 Analytic strategy

Regarding evaluation metrics, Pearson correlation indexed the rank-order associations between model outputs and BFI-2 labels. The *R*^2^ statistic indexed predictive fit relative to the sample mean benchmark; negative *R*^2^ indicated worse performance than predicting the sample mean for all cases. RMSE and MAE indexed error magnitude. Lower RMSE or MAE values were not automatically interpreted as indicative of better measurement because such gains could arise from center shrinkage.

Signed error (predicted − observed score) was examined for HAR to determine whether direct scoring systematically elevated low scorers or suppressed high scorers. The standardized signed error d expressed directional residual bias in SD units. A model with a positive correlation but a negative *R*^2^ value might preserve some rank ordering while failing to recover the observed score scale, whereas a model with a lower MAE might appear preferable even if it achieved that reduction by suppressing high or low scores. These cases are especially impactful when the criterion distribution is skewed.

Regarding resampling and confidence intervals, for LIWC, CSEM, and HAR, 95% confidence intervals and paired differences were estimated using participant-level bootstrap resampling where participant-level predictions were available or reconstructable. Paired differences were summarized as Δ*r*, Δ*R*^2^, ΔMAE, and ΔRMSE. HCIR values were retained at the archived approach summary level, Fisher-*z* confidence intervals were used for HCIR correlations, and calibration was interpreted from the retained *R*^2^ and RMSE summaries.

In cross-validation procedures, LIWC, CSEM, and HAR used five shuffled K-fold splits with a fixed random seed of 42. For CSEM, archived participant-level out-of-fold predictions were reconstructed from the archived PCA feature matrices and the original fivefold RidgeCV workflow. For HAR, although the scoring itself was zero-shot without supervised parameter fitting, we applied the same fivefold splits to ensure that HAR’s evaluation metrics were computed on comparable partitions as the other methods. Specifically, the folds were used only for partitioning the evaluation set—the HAR scores themselves were unaffected by the split—and the same participant-level bootstrap resampling was applied across all approaches to maintain comparability in confidence interval estimation. We therefore describe HAR’s cross-validation as serving harmonization and evaluation rather than model fitting. HCIR used nested cross-validation. The outer evaluation loop used five shuffled K-fold splits with the same fixed random seed of 42, and we concatenated out-of-fold predictions across test folds before computing the final *r*, *R*^2^, and RMSE values. Within each training fold, GridSearchCV (scikit-learn version 1.3.0) used four shuffled K-fold splits and negative mean squared error to tune ElasticNet, support vector regression (SVR), HistGradientBoostingRegressor, and partial least squares (PLS) pipelines. These models were selected to cover linear (ElasticNet, PLS), kernel-based (SVR), and tree-based (HistGradientBoosting) approaches.

Hyperparameter search ranges for the HCIR nested cross-validation were as follows: ElasticNet used l1_ratio = [0.1, 0.5, 0.9] and alpha = logspace(−3, 3, 20); SVR used C = logspace(−2, 3, 10), epsilon = [0.01, 0.1, 0.5], and kernel = [‘linear’, ‘rbf’]; HistGradientBoostingRegressor used learning_rate = [0.01, 0.1, 0.2], max_iter = [100, 200, 300], and max_depth = [3, 5, 10]; PLS used n_components = range(5, 50, 5). All experiments were conducted on a Linux server (Ubuntu 22.04) with 64 GB RAM and an NVIDIA A100 GPU (for LLM inference). Full details of the implementation are provided in Supplementary Materials.

Regarding statistical power, observed sensitivity calculations contextualized the sample size. With *N* = 374, a two-tailed correlation test at *α* = 0.05 had approximately 80% power for effects of approximately |*r*| = 0.14 and 90% power for effects of approximately |*r*| = 0.17. Therefore, the study was sensitive to small-to-moderate effects, although estimates near |*r*| = 0.10 remained weak and comparatively imprecise.

## 3 Results

### 3.1 Prompt-level characteristics

The five prompts differed in response length, missingness, and self-relevance classification (Table 3). The work prompt produced the longest mean responses on average (180.18 ± 86.63 characters), followed by the family and friends prompts. Missingness was negligible except for the disliked-character prompt, which had 18 missing responses (4.8%).

The self-relevance summaries underscored the distinction between self-projection and evaluation prompts. Direct-self responding was high for the work, family, and friendship prompts, whereas the preferred- and disliked-character prompts were dominated by idealized or low-self responding. These descriptors originated from the archived Stage 1 pipeline and therefore represent internal cue summaries as opposed to independent human-coded annotations. Nevertheless, they clarify why the task should not be treated as comprising five interchangeable personality items.

### 3.2 Overall comparison across approaches

Table 4 summarizes the approach-level performance, and Figure 1 displays the mean absolute Pearson correlations across the five domains. LIWC showed a weak association overall (mean |*r*| = 0.079). CSEM showed a modest improvement (mean |*r*| = 0.118; mean *R*^2^ = 0.011), with its clearest gain being for Extraversion. HAR produced a stronger rank-order association than LIWC and CSEM, but its mean *R*^2^ value was negative in both variants. In the retained comparison, HCIR-Grounded showed the most balanced association–calibration profile (mean |*r*| = 0.264, mean *R*^2^ = 0.073, mean RMSE = 1.833), followed by HCIR-Sensitive (mean |*r*| = 0.231, mean *R*^2^ = 0.037, mean RMSE = 1.865).

**Table 4:** Approach-level comparison of association and calibration metrics, including the inferential basis for each approach.

Approach	Evidence representation	Primary implementation	Mean |*r*|	Mean *R*^2^	Mean RMSE	Inference basis
LIWC	Lexical categories	LIWC + RidgeCV	0.079	−0.005	1.910	Participant-level out-of-fold predictions with bootstrap intervals
CSEM	Contextual semantic embeddings	BGE-M3 + Qwen3 embeddings + RidgeCV	0.118	0.011	1.894	Reconstructed participant-level out-of-fold predictions with bootstrap intervals
HAR-discriminative	Direct LLM trait scoring	OpenAI GPT-5.4	0.198	−0.355	2.200	Participant-level direct scores with bootstrap intervals
HAR-conservative	Direct LLM trait scoring	DeepSeek 3.2	0.167	−0.174	2.055	Participant-level direct scores with bootstrap intervals
HCIR-grounded	Cue integration + supervised calibration	DeepSeek 3.2	0.264	0.073	1.833	Retained approach-level summary with Fisher*-z* intervals for *r*
HCIR-Sensitive	Cue integration + supervised calibration	OpenAI GPT-5.4	0.231	0.037	1.865	Retained approach-level summary with Fisher-*z* intervals for *r*

LIWC = Linguistic Inquiry and Word Count lexical baseline; CSEM = contextual semantic embeddings; HAR = holistic appraisal route based on direct large-language-model scoring; HCIR = hierarchical cue integration route. CSEM participant-level effect sizes were reconstructed from archived PCA feature matrices and the original fivefold RidgeCV workflow. HCIR values are retained as approach-level summaries, with Fisher-z confidence intervals for *r*.

**Figure 1:**
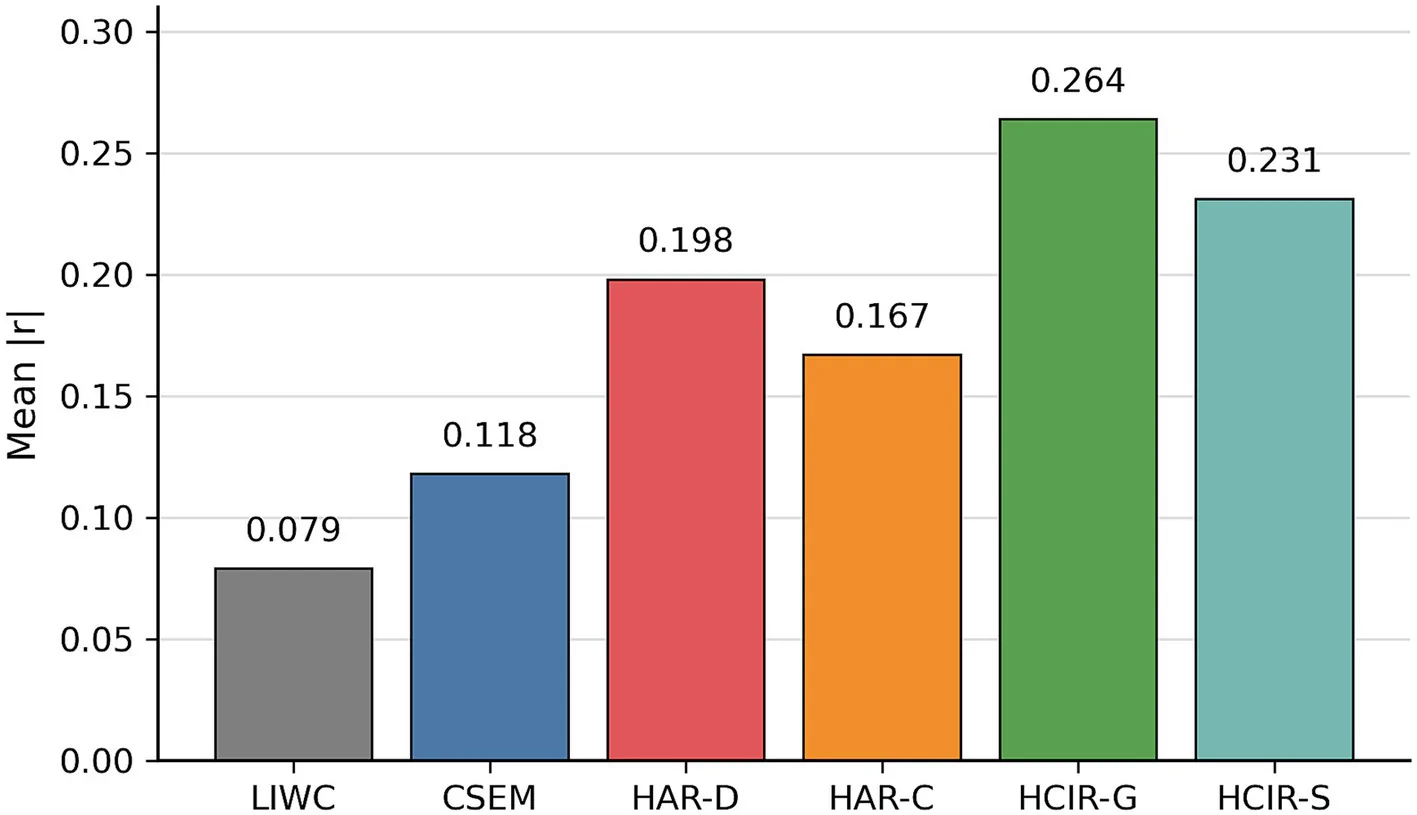
Mean absolute correlations across scoring variants. Bars show the mean |*r*| across the five Big Five domains. Higher values indicate stronger rank-order correspondence with the questionnaire labels.

The association–calibration contrast is central to evaluating these approaches. HAR-Discriminative and HAR-Conservative increased the mean absolute correlations relative to CSEM, but their negative mean *R*^2^ values indicated poor scale recovery. The lower correlations for CSEM were accompanied by modestly positive mean *R*^2^ values. The improvement for CSEM over LIWC was most evident for Extraversion, where the reconstructed participant-level comparison showed *r* = 0.255 and *R*^2^ = 0.063. Paired bootstrap estimates from the archived analysis indicated a reliable LIWC-to-CSEM improvement for Extraversion, whereas the semantic gains for the remaining traits were smaller and less stable. In the archived HCIR comparison, the combination of cue organization and supervised calibration was associated with a more favorable balance between rank-order association and calibration, particularly for Agreeableness, Conscientiousness, and Neuroticism.

### 3.3 Trait-level recoverability

The trait-level correlations are shown in Table 5 and Figure 2. Extraversion was the most recoverable domain across all the approaches. The correlations were 0.085 for LIWC, 0.255 for CSEM, 0.461 for HAR-Discriminative, 0.466 for HAR-Conservative, 0.442 for HCIR-Grounded, and 0.459 for HCIR-Sensitive. This pattern of results supports the interpretation that the task made socially visible self-positioning comparatively available, and it also shows why the evaluation cannot be reduced to a single overall mean. A method performing adequately for Extraversion might still fail for less visible or more evaluatively loaded domains, and a task that elicits strong social positioning cues might not provide the same bandwidth for emotional vulnerability, imaginative exploration, or private self-regulation.

**Table 5:** Trait-level correlations across the lexical, semantic, direct-scoring, and cue integration approaches.

Method	Extraversion	Agreeableness	Conscientiousness	Neuroticism	Openness	Mean |*r*|
LIWC	0.085	−0.058	0.109	0.060	−0.084	0.079
CSEM	0.255	0.122	0.035	0.075	0.103	0.118
HAR-discriminative	0.461	0.175	0.091	0.129	0.136	0.198
HAR-conservative	0.466	0.178	0.036	0.045	0.111	0.167
HCIR-grounded	0.442	0.236	0.289	0.199	0.152	0.264
HCIR-sensitive	0.459	0.128	0.185	0.227	0.158	0.231

Values are Pearson correlations between predicted trait scores and BFI-2 domain labels. Mean |*r*| is the mean absolute correlation across the five Big Five domains.

**Figure 2:**
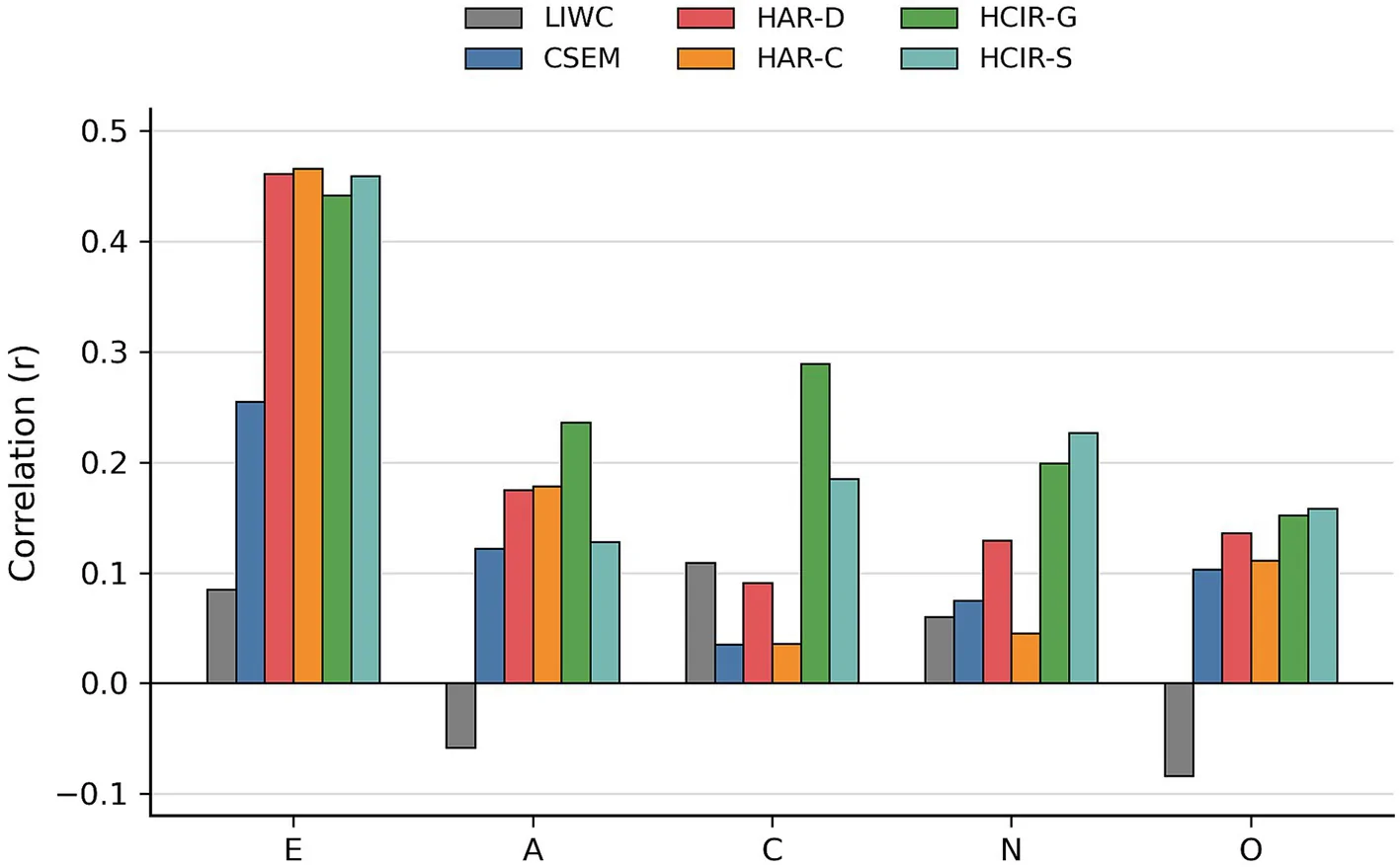
Trait-level correlations across the lexical, semantic, direct-scoring, and cue integration approaches. E = extraversion; A = agreeableness; C = conscientiousness; N = Neuroticism; O = openness.

Agreeableness, Conscientiousness, and Neuroticism exhibited weaker and more method-dependent recoverability. Agreeableness was highest in HCIR-Grounded (0.236), followed by LIWC, CSEM, and HAR. Conscientiousness showed the highest observed correlation in HCIR-Grounded (0.289) despite low correlations from HAR and CSEM. Neuroticism remained modest but showed higher observed correlations in HCIR relative to LIWC, CSEM, and HAR-Conservative. Openness was weak across all methods, with correlations of 0.056–0.158. Therefore, low Openness recoverability should be interpreted as a substantive task fit limitation rather than as a hidden strong prediction that current methods failed to uncover.

### 3.4 Direct scoring: rank-order information and calibration Bias

Table 6 and Figure 3 reveal a dissociation between rank-order preservation and scale calibration in HAR. HAR-Discriminative achieved a positive *R*^2^ value only for Extraversion (0.073); for Agreeableness, Conscientiousness, Neuroticism, and Openness, *R*^2^ was negative. HAR-Conservative followed the same pattern: a positive *R*^2^ value for Extraversion (0.104) but negative values elsewhere. Despite exhibiting lower MAE and RMSE than HAR-Discriminative across all five traits, HAR-Conservative achieved this reduction through stronger central compression—a phenomenon examined in detail below.

**Table 6:** Trait-level association and calibration metrics for HAR and HCIR variants.

Approach	Trait	*r*	*R* ^2^	RMSE
HAR-discriminative	Extraversion	0.461	0.073	1.929
HAR-discriminative	Agreeableness	0.175	−0.393	2.341
HAR-discriminative	Conscientiousness	0.091	−0.470	2.264
HAR-discriminative	Neuroticism	0.129	−0.689	2.290
HAR-discriminative	Openness	0.136	−0.296	2.176
HAR-conservative	Extraversion	0.466	0.104	1.898
HAR-conservative	Agreeableness	0.178	−0.262	2.225
HAR-conservative	Conscientiousness	0.036	−0.272	2.107
HAR-conservative	Neuroticism	0.045	−0.312	2.015
HAR-conservative	Openness	0.111	−0.128	2.032
HCIR-grounded	Extraversion	0.442	0.195	1.797
HCIR-grounded	Agreeableness	0.236	0.039	1.945
HCIR-grounded	Conscientiousness	0.289	0.072	1.804
HCIR-grounded	Neuroticism	0.199	0.037	1.733
HCIR-grounded	Openness	0.152	0.023	1.888
HCIR-sensitive	Extraversion	0.459	0.211	1.782
HCIR-sensitive	Agreeableness	0.128	0.002	1.979
HCIR-sensitive	Conscientiousness	0.185	−0.054	1.919
HCIR-sensitive	Neuroticism	0.227	0.024	1.741
HCIR-sensitive	Openness	0.158	0.001	1.906

A positive *R*^2^ value indicates better performance than the sample mean benchmark; a negative *R*^2^ value indicates worse performance than that benchmark. RMSE is reported on the 1–9 criterion-score metric.

**Figure 3:**
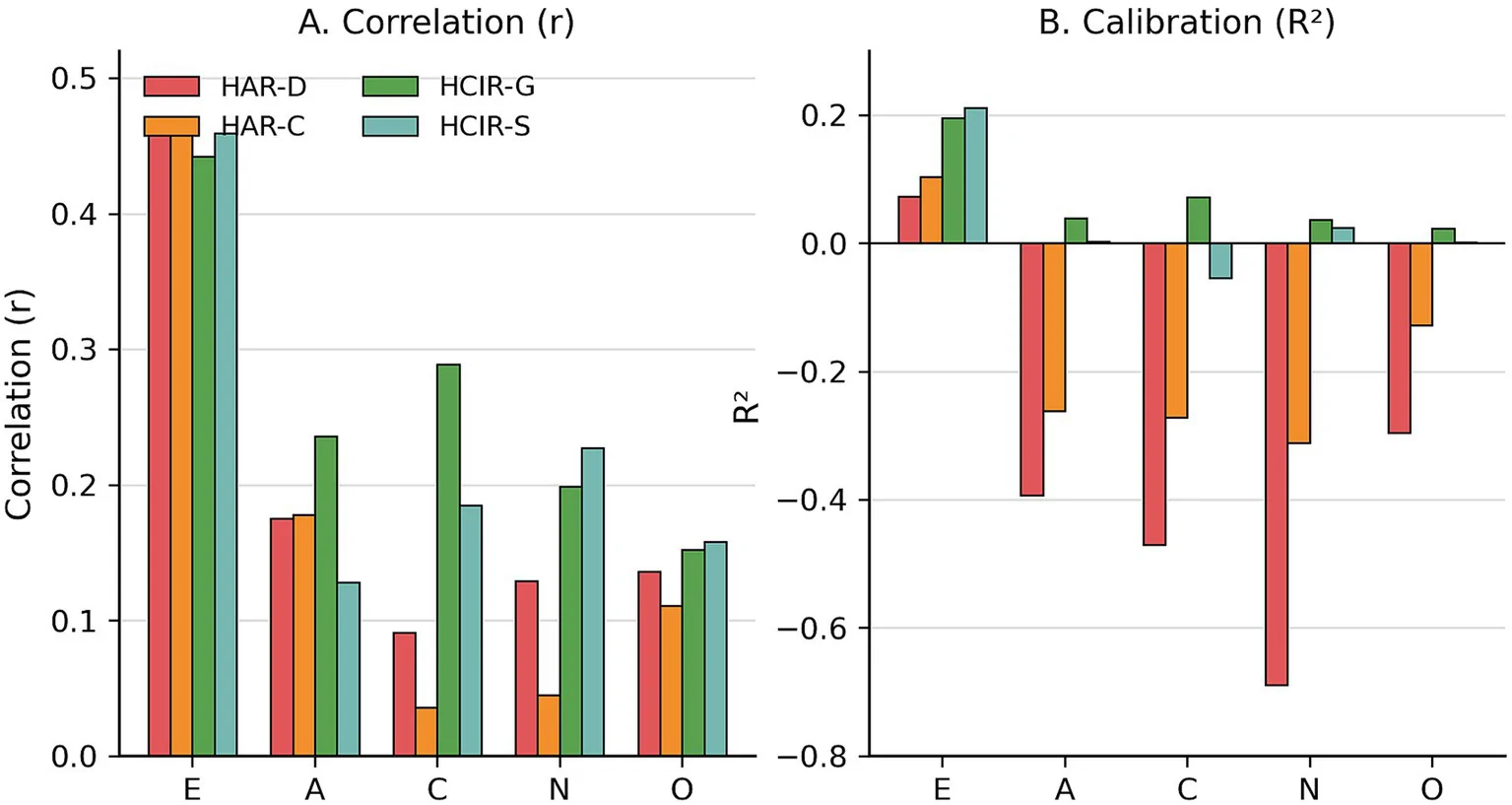
Correlation and calibration diverged across the direct and cue-based approaches. **(A)** Pearson correlations. **(B)** *R*^2^ values relative to the sample mean benchmark. A positive *R*^2^ value indicates successful scale recovery beyond the benchmark; negative *R*^2^ indicates calibration failure despite any rank-order information.

These findings should not be interpreted as evidence that direct scoring captures no personality-relevant information, instead showing that HAR preserves rank-order information sufficient for the relative ordering of individuals while failing to recover the absolute metric of the questionnaire scale. Extraversion stands apart as the only domain for which direct scoring achieved both reasonable rank-order association and positive *R*^2^ values. The negative *R*^2^ values for Agreeableness, Conscientiousness, Neuroticism, and Openness indicate a systematic calibration failure; whether *post hoc* calibration (e.g., linear rescaling or isotonic regression) could mitigate these biases remains untested and is a direction for future work.

### 3.5 Signed error and central compression in HAR

Table 7 and Figure 4 display the signed error in the most distorted extreme score bands. Both HAR variants overestimated low Agreeableness and low Conscientiousness and underestimated high Neuroticism and high Openness. For example, HAR-Conservative produced standardized signed error effects of *d* = 3.95 for low Agreeableness, *d* = 2.60 for low Conscientiousness, *d* = −2.78 for high Neuroticism, and *d* = −1.88 for high Openness. The corresponding HAR-Discriminative values were *d* = 3.15, *d* = 2.65, *d* = −1.39, and *d* = −1.25.

**Table 7:** Signed error patterns and standardized residual bias effect sizes for HAR predictions in the most distorted extreme score bands.

Model	Trait	Band	*N*	Observed M	Predicted M	Signed error	Std. signed error d	*d* 95% CI
HAR-conservative	Agreeableness	Low (≤3)	38	2.394	6.668	4.274	3.950	[3.021, 5.713]
HAR-conservative	Conscientiousness	Low (≤3)	35	2.229	6.114	3.886	2.597	[2.206, 3.302]
HAR-conservative	Neuroticism	High (≥7)	26	7.654	4.138	−3.515	−2.781	[−4.525, −1.983]
HAR-conservative	Openness	High (≥7)	146	7.760	5.618	−2.142	−1.878	[−2.146, −1.666]
HAR-discriminative	Agreeableness	Low (≤3)	38	2.394	6.821	4.427	3.146	[2.346, 4.787]
HAR-discriminative	Conscientiousness	Low (≤3)	35	2.229	6.474	4.246	2.651	[2.109, 3.621]
HAR-discriminative	Neuroticism	High (≥7)	26	7.654	5.315	−2.338	−1.387	[−2.084, −0.979]
HAR-discriminative	Openness	High (≥7)	146	7.760	5.888	−1.873	−1.246	[−1.466, −1.069]

Signed error is defined as predicted − observed. Positive values indicate overprediction, and negative values indicate underprediction. The standardized signed error d represents the directional residual bias in standard deviation units. The confidence intervals are participant-level bootstrap intervals for *d*.

**Figure 4:**
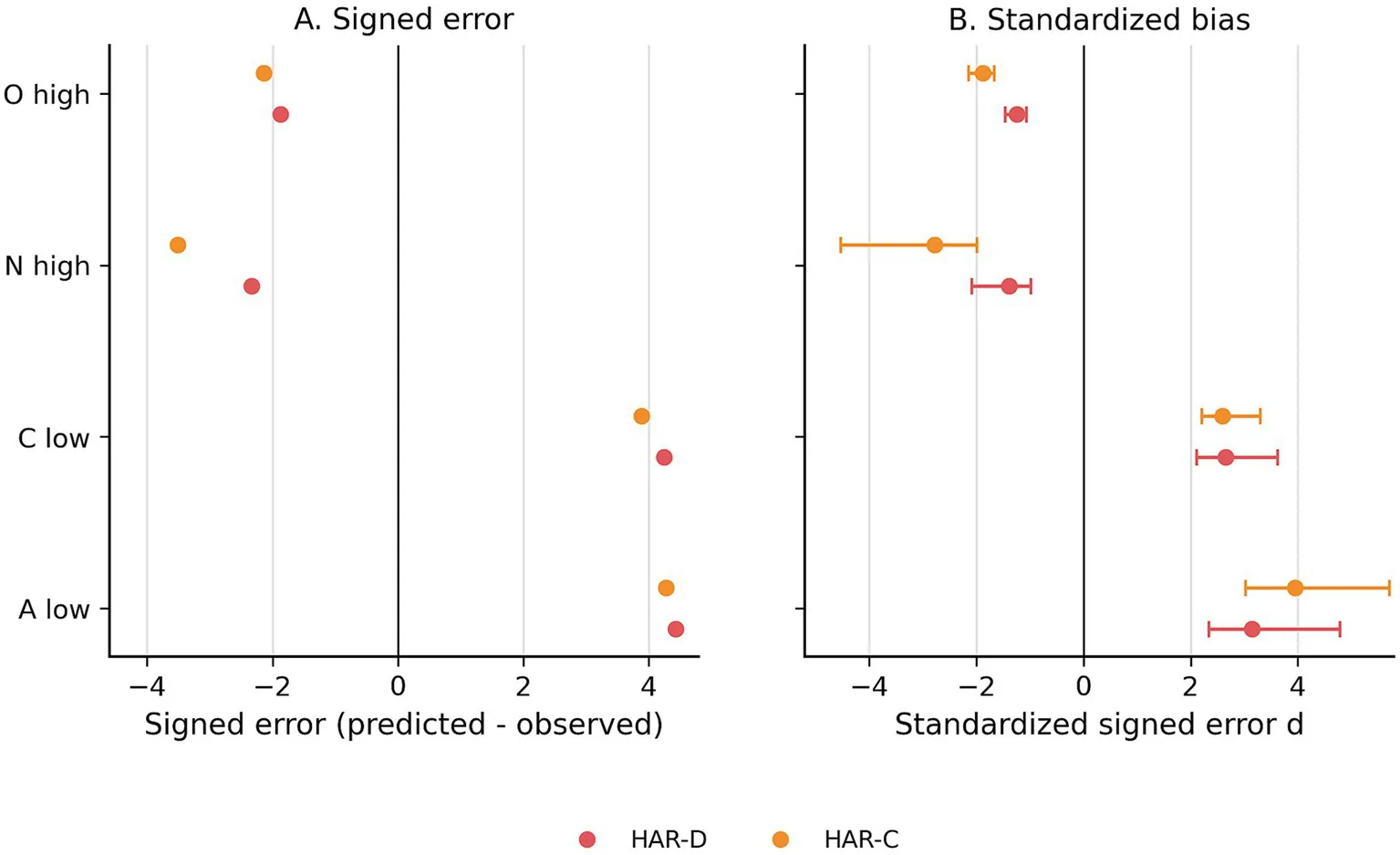
Signed error patterns in the extreme score bands of HAR predictions. Panel **(A)** shows the mean signed error, defined as predicted − observed. Panel **(B)** shows the standardized signed error, *d*, with bootstrap confidence intervals. Positive values indicate overprediction, and negative values indicate underprediction.

These biases are both psychologically directional and theoretically interpretable. Direct scoring pulled low Agreeableness and low Conscientiousness upward toward normatively acceptable levels, while also pushing high Neuroticism and high Openness downward. This pattern is consistent with a central compression bias—or, equivalently, an “acceptable center” bias—in which direct LLM judgments avoid extreme scores and regress toward the normative mean.

This finding has important implications for evaluating predictive models in personality assessment. Although a model that achieves lower average error (MAE or RMSE) by shrinking extreme predictions toward the center might appear superior according to conventional accuracy metrics, this apparent improvement obfuscates a systematic distortion of the score scale. In applied contexts, such bias is important: low prosociality, low self-regulation, high emotional distress, and unusually high openness have distinct psychological and behavioral implications that differ qualitatively from moderate scores. Although a system that systematically attenuates these extremes might appear cautious or conservative, it reduces the discriminative meaning of the scale.

### 3.6 Internal variation within HCIR

The two HCIR variants differed in both output profile and cue-harvesting summaries (Table 8; Figure 5). HCIR-Grounded achieved correlations of 0.442, 0.236, 0.289, 0.199, and 0.152 for Extraversion, Agreeableness, Conscientiousness, Neuroticism, and Openness, respectively, whereas HCIR-Sensitive achieved respective correlations of 0.459, 0.128, 0.185, 0.227, and 0.158. The sensitive branch was slightly higher for Extraversion, Neuroticism, and Openness, whereas the grounded branch was higher for Agreeableness and Conscientiousness.

**Table 8:** Internal comparison of HCIR variants across output and cue-harvesting summaries.

HCIR variant	Mean |*r*|	Valid prompts M	Clear prompts M	Domain coverage M	Insufficient traits M	Quality cues M	Direct-self prompts M	Q7/Q8 idealized-low-self M	Needs review (%)
HCIR-Grounded	0.264	4.925	4.863	9.247	0.153	5.531	2.826	1.692	0.8%
HCIR-Sensitive	0.231	4.952	4.762	10.005	0.059	5.297	2.639	1.612	2.9%

M = mean. Domain coverage reflects the average breadth of trait-relevant evidence extracted across prompts. Quality cues refer to retained quality-related cues. Direct-self prompts are prompts classified as directly self-referential in the archived Stage 1 pipeline. Needs review % is the proportion of cases flagged by the archived extraction workflow for potential quality concerns.

**Figure 5:**
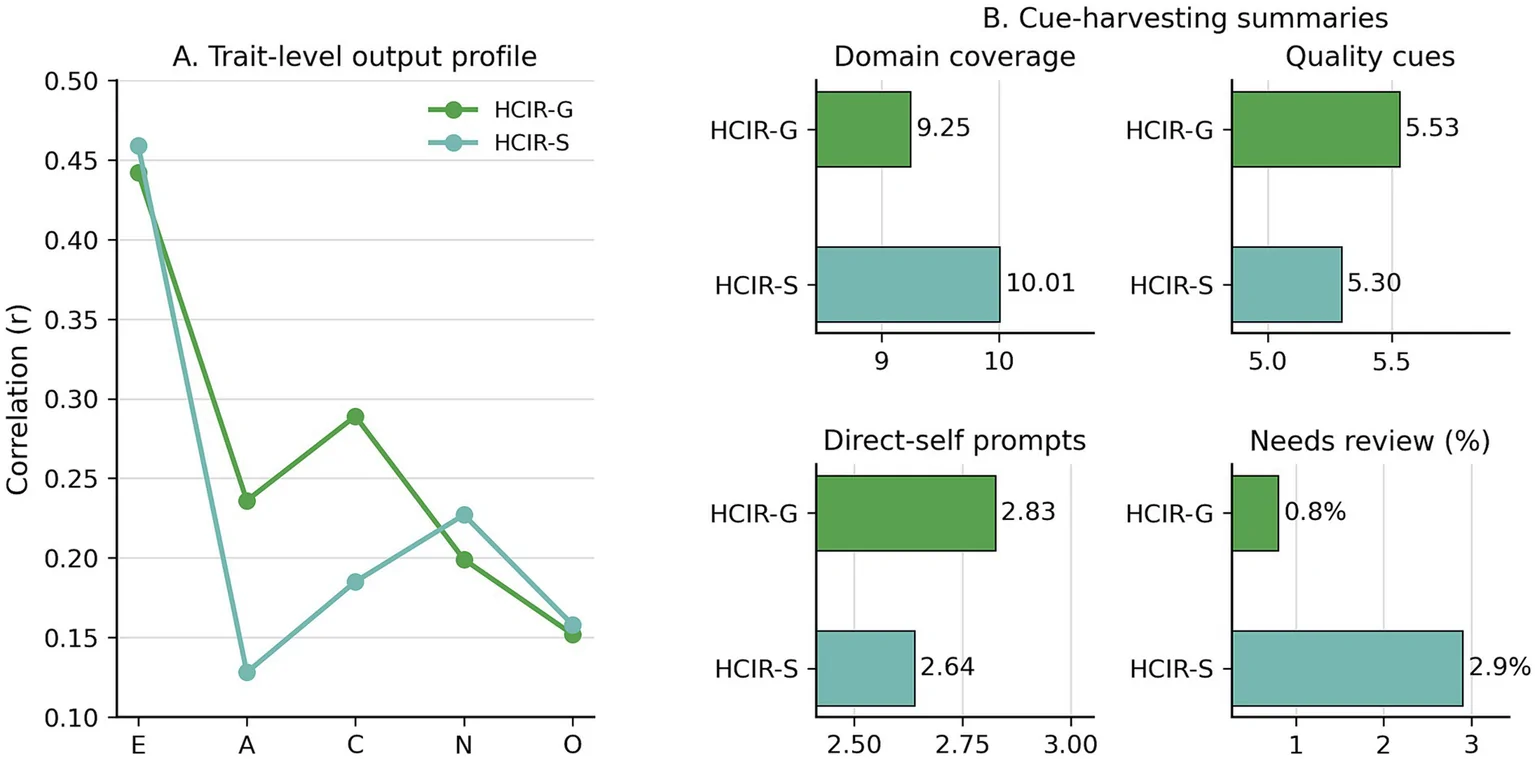
HCIR variants differed in both output profile and evidence harvesting. Panel **(A)** compares Pearson correlations as trait-level outputs. Panel **(B)** presents metric-specific cue-harvesting summaries on their native scales; values should be interpreted within each subpanel rather than compared across subpanels.

Calibration outcomes followed a similar pattern of divergence. HCIR-Grounded yielded positive *R*^2^ values across all five traits. HCIR-Sensitive performed strongest on Extraversion and remained positive for Neuroticism, but it approached zero for Agreeableness and Openness and fell below zero for Conscientiousness.

The cue-harvesting summaries point to a systematic trade-off between breadth of extraction and grounding in the context of self-referential content. HCIR-Sensitive extracted broader domain coverage and produced fewer “insufficient traits” flags, indicating that its extraction stage was more permissive or comprehensive. By contrast, HCIR-Grounded retained more directly self-referential prompts, slightly more quality cues, and a lower proportion of cases flagged for review. This pattern cautions against assuming that broader, or more extensive, cue extraction always improves measurement; instead, the cue-harvesting strategy–downstream predictive performance relationship appears trait-specific and contingent on the degree of grounding in self-descriptive content.

### 3.7 Cue scores as an intermediate layer

The cue-level analyses addressed whether the HCIR cue layer captured information empirically distinct from direct LLM scoring. Table 9 and Figure 6 present three key findings: structured cue scores correlated highly with direct model ratings (cue-direct *r* of 0.076–0.801 across traits and branches), indicating that the extracted cues were not independent of direct LLM judgments; cue scores were not numerically interchangeable with final HCIR outputs, despite these associations, with the latter undergoing supervised calibration that substantially altered the mapping to questionnaire labels; and cue-label correlations were consistently lower than final HCIR correlations, demonstrating that the supervised reweighting step contributed meaningful adjustment beyond the raw cue representations.

**Table 9:** Associations among cue scores, direct model ratings, and criterion labels within HCIR.

HCIR branch	Direct comparator	Trait	*N*	Cue-direct *r*	Cue-label *r*	Direct-label *r*
Grounded cue layer	HAR-conservative	Extraversion	372	0.705	0.410	0.465
Grounded cue layer	HAR-conservative	Agreeableness	372	0.511	0.180	0.177
Grounded cue layer	HAR-conservative	Conscientiousness	372	0.389	0.067	0.036
Grounded cue layer	HAR-conservative	Neuroticism	372	0.076	0.107	0.046
Grounded cue layer	HAR-conservative	Openness	372	0.213	0.084	0.111
Sensitive cue layer	HAR-discriminative	Extraversion	374	0.801	0.446	0.461
Sensitive cue layer	HAR-discriminative	Agreeableness	374	0.525	0.185	0.175
Sensitive cue layer	HAR-discriminative	Conscientiousness	374	0.472	0.073	0.091
Sensitive cue layer	HAR-discriminative	Neuroticism	374	0.533	0.108	0.129
Sensitive cue layer	HAR-discriminative	Openness	374	0.331	0.075	0.136

Cue-direct *r* denotes the correlation between cue layer scores and direct LLM ratings. Cue-label *r* denotes the correlation between cue layer scores and BFI-2 labels. Direct-label *r* is the corresponding direct-scoring correlation for the same branch.

**Figure 6:**
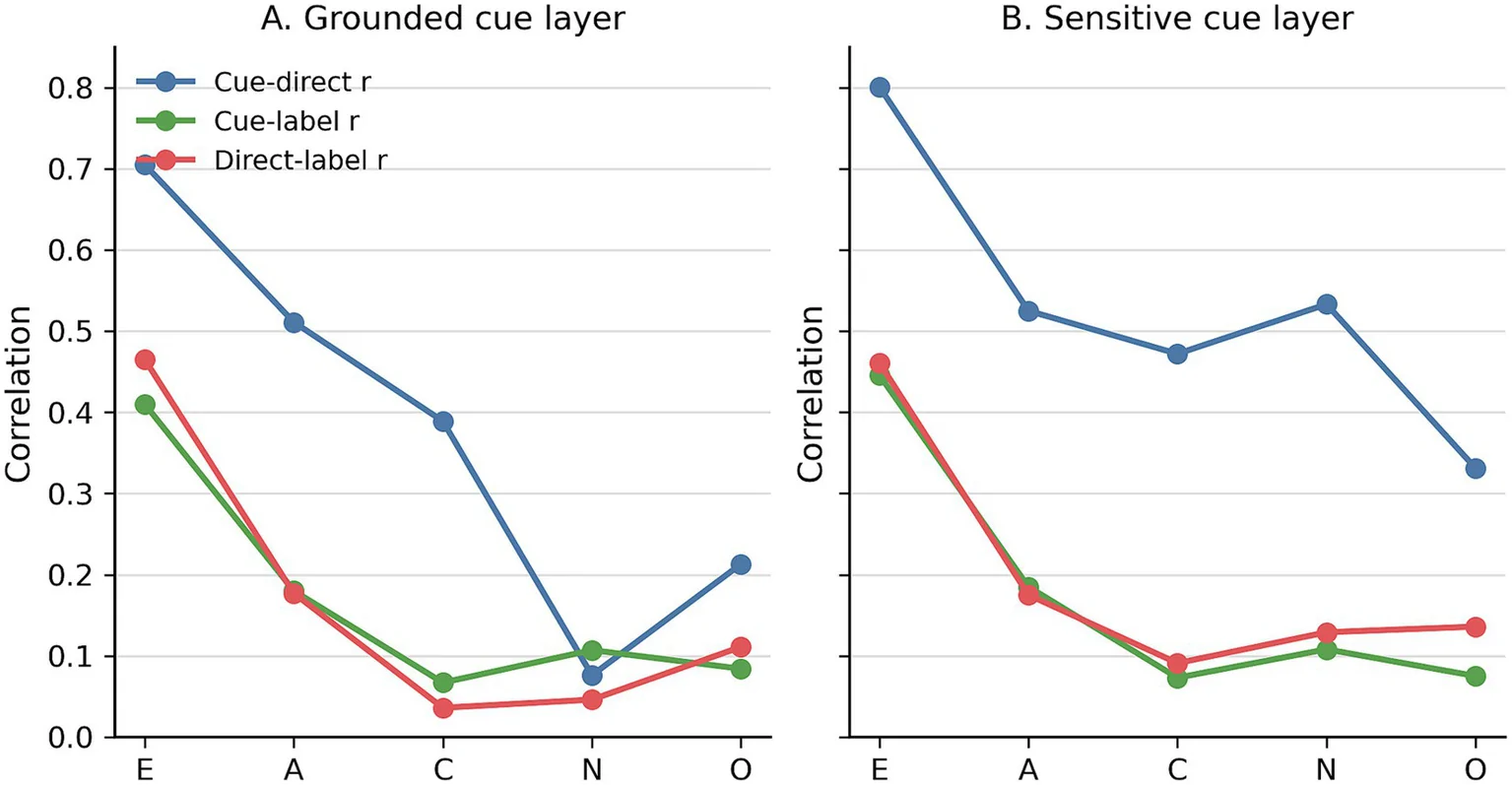
Cue layer scores were related to direct ratings but were not interchangeable with the final HCIR outputs. **(A)** Grounded cue layer. **(B)** Sensitive cue layer. The figure compares associations among cue scores, direct ratings, and questionnaire labels, illustrating that the cue layer served as intermediate evidence rather than as a final score.

This pattern of results argues against interpreting HCIR as merely a relabeled version of direct scoring: the cue layer preserved information related to direct LLM impressions while not remaining reducible to those impressions or the final calibrated scores. Therefore, the intermediate representation played a genuine mediational role in the prediction pipeline rather than functioning as a superficial recoding of direct model outputs.

Table 10 lists selected theory-consistent cue-label associations derived from the two V7 cue pipelines. These correlations are not substitutes for the final HCIR outputs but rather evidence that the cue layer captured psychologically interpretable variance. The most consistent cue-level evidence emerged for Extraversion: positive Extraversion cue means correlated positively with BFI-2 Extraversion, whereas limiting Extraversion cue means had negative correlations. Selected cues for Agreeableness, Conscientiousness, and Openness also showed the expected directions, including boundary consideration (positive), self-centered relational stance (negative), checking and sequencing (positive), task avoidance (negative), imaginative inference (positive), and early closure (negative).

**Table 10:** Selected theory-consistent correlations between concrete HCIR cue indicators and BFI-2 domain labels.

Branch	Trait label	Cue indicator	*N*	*r* [95% CI]
Grounded	Extraversion	Positive Extraversion cue mean	324	0.141 [0.035, 0.233]
Grounded	Extraversion	Limiting Extraversion cue mean	242	−0.225 [−0.334, −0.096]
Sensitive	Extraversion	Positive Extraversion cue mean	340	0.311 [0.218, 0.397]
Sensitive	Extraversion	Limiting Extraversion cue mean	289	−0.338 [−0.441, −0.228]
Sensitive	Agreeableness	AG2: boundary consideration	149	0.195 [0.043, 0.337]
Sensitive	Agreeableness	AGN1: self-centered relational stance	32	−0.322 [−0.563, 0.004]
Sensitive	Conscientiousness	CO2: checking and sequencing	112	0.179 [−0.021, 0.359]
Grounded	Conscientiousness	CON1: task avoidance	21	−0.462 [−0.799, −0.037]
Sensitive	Openness	OP5: imaginative inference	108	0.182 [0.001, 0.362]
Sensitive	Openness	OPN1: early closure	192	−0.192 [−0.350, −0.025]

Grounded refers to the 0412 V7 cue layer, and Sensitive refers to the 0413 V7 GPT-5.4 cue layer. The Positive Extraversion cue mean summarizes active approach, expressive presence, and familiar-context expressiveness, whereas the Limiting Extraversion cue mean summarizes low exposure and peripheral participation. AG2 indexes boundary consideration, AGN1 self-centered relational stance, CO2 checking and sequencing, CON1 task avoidance, OP5 imaginative inference, and OPN1 early closure. Confidence intervals are participant-level bootstrap intervals. Rows with sparse cue occurrence, especially AGN1 and CON1, should be interpreted as directional cue evidence rather than stable, standalone scale estimates.

These cue-level results support the plausibility of the intermediate representation but do not validate standalone microscales. Several caveats warrant further explication. First, many cue indicators occurred sparsely, limiting the stability of their correlational estimates. Second, the cue layer was generated by an LLM pipeline rather than independent human coding; therefore, the reported associations reflect model-derived regularities rather than human-consensus judgments. Third, the theory-consistent directions observed for selected cues should be interpreted as exploratory evidence consistent with the proposed framework rather than as confirmatory tests of specific cue-level hypotheses. Future work should apply human validation to these cue indicators and test their generalizability across tasks and populations.

## 4 Discussion

### 4.1 Main findings

This study compared four approaches—LIWC, CSEM, HAR, and HCIR—for predicting Big Five domains from spoken responses to a picture-based self-projection task. Four findings stand out, as follows.

First, the five prompts were not psychologically equivalent. The work, family, and friendship prompts primarily elicited self-projection, whereas the preferred- and disliked-character prompts elicited preference, aspiration, rejection, and normative evaluation. This heterogeneity underpinned what type of evidence was available for downstream prediction.

Second, trait recoverability differed substantially across domains: Extraversion was consistently the most recoverable trait across all four approaches, whereas Openness was the least recoverable.

Third, HAR preserved rank-order information, particularly for Extraversion, but did not recover scale structure outside that domain. Negative mean *R*^2^ values and systematic signed error biases indicated poor calibration even when the rank-order association appeared adequate.

Fourth, the retained HCIR comparison suggested a more balanced profile, achieving positive mean *R*^2^ values and stronger mean absolute correlations, with notably higher observed correlations for Agreeableness, Conscientiousness, and Neuroticism. HCIR also provided inspectable, intermediate evidence, making its predictions more transparent than end-to-end direct scoring.

These findings should not be considered a general demonstration that any particular model family is superior. Rather, they raise a narrower measurement question: when a spoken response task mixes grounded self-description with preference and normative evaluation, how should trait-relevant evidence be represented before scoring? The results suggest that preserving cue provenance, self-relevance, cue quality, and trait-specific content, in conjunction with supervised calibration, may contribute to a more favorable association–calibration profile and improved inspectability. However, because cue extraction and supervised calibration were not independently manipulated, we interpret this as approach-level evidence rather than causal evidence for cue integration per se.

This study therefore contributes both at the framework level and empirically, demonstrating that a language-based personality task can be evaluated not only according to average prediction accuracy but also the psychological status of the cues entering the score. As such, it shifts the evaluation criterion from purely predictive performance toward a more measurement-oriented framework that prioritizes interpretability and calibration alongside rank-order association.

### 4.2 Prompt ecology and trait recoverability

The task was well-suited to Extraversion because it repeatedly allowed socially visible self-positioning; respondents described how they located themselves in work, family, and friendship scenes, creating opportunities to mention participation, approach, expressiveness, boundaries, and interpersonal engagement. These cue types are precisely those that tend to be available in brief person perception and behavioral trace settings (Borkenau and Liebler, 1992; Back et al., 2010; Wu et al., 2015). Extraversion cues showed the most consistent theory-aligned associations in our cue-level analyses, supporting the interpretation that the task captured observable social behavior rather than merely internal states.

Agreeableness and Conscientiousness were more vulnerable to normative tone. Although prosocial stance, boundary consideration, reliability, checking, and role responsibility are meaningful behavioral cues, in a workplace training setting, they can also reflect socially desirable self-presentation rather than stable trait standing. Therefore, direct LLM scoring might conflate prosocial or diligent language with underlying dispositional tendencies. HCIR was useful in separating narrower behavioral meanings from generally favorable language before supervised calibration, allowing the model to learn which cues predicted criterion variance beyond mere normative desirability.

Neuroticism was constrained by both task design and assessment context: respondents could describe conflict, danger, or disliked behavior without disclosing their own anxiety, vulnerability, or emotional instability. The workplace training context might have further discouraged explicit distress-related self-disclosure because participants were likely motivated to present themselves as emotionally stable and professionally composed, with this dual constraint helping to explain why direct scoring systematically compressed high Neuroticism scores—and why the Neuroticism cue associations were weak and inconsistent across methods.

Openness faced a qualitatively different limitation: the fixed picture and role selection prompts permitted some imaginative inference and resistance to early closure but did not strongly invite aesthetic appreciation, intellectual curiosity, abstract reasoning, or exploratory elaboration. Therefore, low Openness recoverability should be interpreted as an expressive bandwidth limitation of this particular task rather than being used to draw a general conclusion about Openness and language. This distinction has important implications for task design. If Openness is a target domain in future research, picture-based tasks will likely need prompts explicitly inviting alternative interpretations, counterfactual narratives, aesthetic preferences, intellectual curiosity, or unconventional perspective taking. Without such opportunities, even a sophisticated scoring model might have little trait-relevant evidence to recover, with prediction remaining at floor regardless of any methodological refinements.

### 4.3 Direct scoring and calibration

Despite its limitations, HAR proved informative because it demonstrated that direct LLM scoring can preserve rank-order information from short, heterogeneous spoken responses. The correlations for Extraversion (*r* ≈ 0.46) were comparable to those in meta-analyses of text-based personality prediction (Koutsoumpis et al., 2022), suggesting that modern LLMs capture socially visible individual differences even from brief task-based speech. However, this rank-order preservation came at the cost of poor calibration.

Outside Extraversion, the mean *R*^2^ value was negative for both HAR variants, indicating that predictions were systematically worse than simply guessing the sample mean. Signed error analyses revealed large directional distortions: low Agreeableness and low Conscientiousness were substantially overestimated, whereas high Neuroticism and high Openness were substantially underestimated. These biases were not marginal; the standardized effect sizes reached *d* = 3.95 for low Agreeableness and *d* = −2.78 for high Neuroticism.

This pattern of results has important implications for LLM-assisted personality assessment; a model can achieve plausible relative ordering of individuals while simultaneously failing to recover the scale metric necessary for questionnaire-level interpretation. Moreover, a model can appear superior in terms of conventional accuracy metrics, such as MAE or RMSE, by shrinking predictions toward the center of the distribution. In asymmetric distributions, where low Neuroticism and high Agreeableness are more common, such center shrinkage is not merely a statistical artifact; it systematically alters the score’s psychological meaning, compressing the extremes that often carry the most diagnostic information. For applied assessment, this is important because individual difference measures are routinely used to distinguish meaningful score regions such as clinical thresholds, selection cutoffs, or developmental risk levels—not only to minimize average error across the full sample (Ozer and Benet-Martínez, 2006; Roberts et al., 2007).

The present comparison did not test whether HAR outputs could be improved through *post hoc* calibration. A stronger design would include multiple conditions: HAR raw scores, HAR plus linear rescaling, HAR plus nonlinear calibration, embedding-based models with matched calibration, cue-only models, cue-plus-quality weighting, and full HCIR. Such an ablation would distinguish the unique contributions of direct LLM judgment, calibration, and cue organization and clarify whether the calibration advantages observed in HCIR arose primarily from the cue extraction stage, the supervised learning stage, or their interaction.

### 4.4 HCIR as measurement-oriented cue integration

HCIR’s contribution is best understood as a measurement-oriented framework rather than as definitive proof of a superior model. Its defining feature is the intermediate cue layer, which explicitly records prompt function, source type, self-relevance, cue quality, domain coverage, and trait-specific evidence before any supervised calibration. This design choice makes the evidentiary structure of the final score more inspectable than an end-to-end direct score. Therefore, HCIR aligns with two foundational traditions in psychological measurement: Brunswikian cue modeling, which emphasizes the ecology of cues available to a judge (Brunswik, 1956), and construct validation logic, which prioritizes articulating theoretical relationships between observations and constructs (Cronbach and Meehl, 1955; Letzring et al., 2006).

The framework also makes error diagnosis more concrete and actionable. When a final score performs poorly, researchers can systematically identify the source of failure by asking questions such as: Did the task fail to elicit relevant cues? Did the LLM-based extractor fail to identify them? Did the cue layer permit too much aspirational or normatively desirable content? Or did the supervised calibration model fail to weight the available evidence appropriately? Each failure mode points to a different remedy, and the cue layer provides the intermediate representation necessary to distinguish among them.

Several limitations of this study must be acknowledged. First, the cue layer was generated by an LLM pipeline, not by independent human raters, so it should not be treated as a validated psychometric scale. Second, our approach-level comparison cannot isolate the independent causal contributions of cue extraction, model family, prompt design, output constraints, and supervised calibration, so the retained HCIR comparison should be interpreted as promising approach-level evidence rather than a definitive model ranking result. Stronger claims would require a fully orthogonal design in which each component is systematically manipulated.

Nevertheless, within these limits, HCIR offers a useful direction for LLM-assisted measurement, reframing the role of the LLM from a final trait scorer to a structured cue extractor with outputs that are subsequently calibrated against criterion labels. This reframing is especially valuable when source texts combine heterogeneous elements such as self-description, aspiration, preference, and moral evaluation, as in our picture-based task. By separating evidence extraction from evidence weighting, HCIR creates a natural opportunity for future human validation. Human raters can evaluate prompt function, cue quality, and trait relevance at the intermediate layer rather than judging only whether the final numeric score approximates a questionnaire label. Such “human-in-the-loop” validation could provide convergent evidence of the psychological meaningfulness of the extracted cues and help identify systematic biases that purely data-driven calibration might miss.

### 4.5 Implications

Our findings have three implications for language-based personality assessment. First, task design and cue ecology should be part of the measurement model. Texts from diaries, social media, interviews, workplace assessments, and picture-based response tasks are not interchangeable input streams; they differ in terms of what they invite people to say and how directly those statements refer to the self. Treating all text as equivalent input can obscure whether a model is detecting stable trait expression, situational role performance, platform convention, moral evaluation, or response-style artifacts–an approach that is especially relevant for short tasks. With only five spoken responses, the measurement burden largely depends on whether the prompts elicit the appropriate type of evidence.

Second, studies should report more than correlations. Though useful, rank-order association does not establish scale recovery. *R*^2^ relative to a sample mean benchmark, RMSE, MAE, signed error, extreme-band bias, and trait-specific recoverability reveal different forms of success and failure; this fact is particularly important for LLM-based scoring, where fluent global impressions could conceal calibration bias. Reporting only correlations would make HAR appear more successful than it was as a scale-recovery method, whereas reporting only MAE or RMSE would obscure whether lower error arose from meaningful prediction or scores shrinking toward the center. A psychometric evaluation should therefore ask both whether people are ordered correctly and whether the predicted scores preserve interpretable locations at the criterion scale.

Third, LLMs might be more suitable as structured cue extractors than as direct trait scorers in heterogeneous open-ended tasks. Direct scoring can be useful, especially for rank-order information, but cue integration allows evidence provenance preservation and makes construct-relevant assumptions inspectable before final prediction. This fact does not mean that cue extraction is automatically valid; rather, cue extraction creates an intermediate object that can be audited, compared with human coding, stress-tested across prompts, and recalibrated when the task or population changes. That intermediate object is valuable precisely because it exposes the assumptions that direct scoring leaves implicit.

## 5 Limitations and future directions

Several design features constrain our conclusions. First, the sample was drawn from offline workplace training sessions; although this setting might have increased ecological relevance for work-related self-projection, it could also have encouraged norm-consistent self-presentation. The results might not generalize to diaries, clinical narratives, social media posts, private writing, or other lower-stakes contexts.

Second, the task order was fixed. Participants completed the BFI-2 questionnaire first, with the spoken picture task following approximately 1 day later. Although the 1-day separation reduces concern about immediate item-level priming relative to same-session administration, broader trait-relevant self-reflection or shared assessment-context effects cannot be fully ruled out; future work should counterbalance task order or separate administrations more systematically.

Third, the stimulus and prompts limited expressive bandwidth. Although the fixed picture supported comparability across respondents, it also constrained the range of imaginative, emotional, intellectual, and autobiographical material that participants could provide. This limitation could be especially relevant to Openness and Neuroticism, where the same design feature that improves standardization can reduce construct coverage. Future studies should consider using multiple stimuli, alternate prompt sets, or adaptive follow-up questions to test whether trait recoverability changes as response opportunity broadens.

Fourth, the analyses used transcripts rather than acoustic signals. Although approximately 10% of the valid participant records (*n* = 37) were evaluated against manually transcribed reference texts and yielded an overall CER of 0.08, transcription errors might still have affected short, ambiguous, or acoustically noisy responses. Moreover, the comparison used transcripts rather than acoustic signals, omitting prosodic features such as pause structure, speech rate, intensity, and vocal affect from the analysis.

Fifth, cue validity remains limited. Self-relevance, source type, quality cues, and trait-specific cues came from the LLM pipeline rather than independent human coding. Future studies should create human-coded benchmarks for prompt function, self-relevance, cue quality, and trait-specific evidence, and should report inter-rater reliability before treating cue layer variables as validated interpretive categories.

Sixth, model comparison and reproducibility were constrained by provenance. Representation, model family, prompt design, output constraints, and calibration method covaried, so HCIR’s retained advantage cannot be attributed only to cue extraction. The final HCIR interpretation rests on retained summary-level comparison files and cue layer records, whereas LIWC, CSEM, and HAR also supported participant-level prediction analyses; this difference affects the strength of paired participant-level inference for HCIR. Future work should preserve frozen prompts, exact model versions, run manifests, intermediate cue records, participant-level out-of-fold predictions, final scripts, and environment details. For LLM-assisted assessment, such provenance is not optional; it is part of the measurement evidence because model outputs vary with prompt wording, model version, decoding settings, and fallback rules.

Seventh, the criterion was concurrent validity against BFI-2 self-report. The study does not establish whether the same pattern would hold for peer reports, supervisor ratings, behavioral criteria, clinical outcomes, or longitudinal adjustment— an important limitation because self-reports, observer reports, and behavioral outcomes can emphasize different aspects of personality. A cue that helps recover self-reported Extraversion might not add the same value for peer-rated sociability or workplace participation, and a cue that predicts self-reported Conscientiousness might not predict supervisor-rated reliability. Future work should test whether cue-integrated representations improve external validity and incremental validity beyond questionnaire scores.

Finally, the comparison was not in the form of a strict causal ablation; a stronger design would compare HAR raw scoring, HAR plus *post hoc* calibration, embeddings plus matched calibration, cue-only models, cue plus quality weighting, and full HCIR while holding the model family and calibration protocol more constant. Such work is necessary before attributing performance gains to any single component. The most informative approach would be to freeze prompts and model versions before data analysis, save every participant-level prediction vector, and preregister the metrics that define success for association, calibration, and interpretability. Such a design would allow the field to distinguish model capacity from measurement discipline.

## 6 Conclusion

This study provides preliminary evidence that, for spoken responses mixing self-projection, preference, and normative evaluation, cue-integrated scoring might offer a more psychometrically disciplined alternative to direct end-to-end LLM scoring. Direct scoring preserved rank-order information but showed substantial calibration bias, whereas the retained HCIR comparison suggested a more balanced association–calibration profile and superior evidentiary inspectability. These findings support treating LLM-assisted personality inference as a measurement problem rather than merely as a prediction problem. Future progress depends on matching prompts to trait-relevant cue ecologies, preserving intermediate evidence, reporting calibration and signed error alongside correlation, and archiving the full participant-level prediction workflow necessary for independent replication.

## Data Availability

The original contributions presented in the study are included in the article/Supplementary material, further inquiries can be directed to the corresponding author.
