# Supplementary material for: From direct scoring to cue integration: big five prediction from spoken responses to a picture-based self-projection task
Source: Front Psychol. 2026 Sep 30;17:1930314. doi: 10.3389/fpsyg.2026.1930314 (PMC13662918; doi:10.3389/fpsyg.2026.1930314)
Supplement: Supplementary file 1 [file Supplementary_file_1.docx]

***Supplementary Material***

# Appendix A: Complete Prompt Texts and Inference Parameters

Table A1. HAR Stage 1: Domain-Level Evidence Extraction Prompt

| Item | Content |
| --- | --- |
| Role | You are a personality psychologist specializing in the Big Five model. |
| Task | Below are five spoken responses from a participant who viewed a picture with numbered characters and answered the following prompts: (1) which character is most like you in a work context and why; (2) which character is most like you in a family context and why; (3) which character is most like you in a friendship context and why; (4) which character do you like most and why; (5) which character do you like least and why. |
| Instruction | Extract the relevant behavioral and psychological evidence for each of the Big Five domains: Extraversion, Agreeableness, Conscientiousness, Neuroticism, and Openness. For each domain, summarize the key evidence from the responses that is relevant to that trait. Be specific and cite the prompt source. |
| Output Format | Provide your output as a bulleted list with five sections, one per trait. |

Table A2. HAR Stage 2: Trait Scoring Prompt

| Item | Content |
| --- | --- |
| Role | You are a personality psychologist specializing in the Big Five model. |
| Task | Based on the extracted evidence provided above, rate this participant's level of [TRAIT] on a 1-to-9 scale, where 1 is extremely low and 9 is extremely high. |
| Instruction | Consider both the presence of trait-relevant behaviors and the absence of counter-indicative behaviors. Use the full range of the scale. Do not compress scores toward the center. Provide only the numeric score. |
| Output Format | A single integer from 1 to 9. |

Note: This prompt was run independently five times per trait (five rating passes). The mean of the five scores was retained for each trait.

Table A3. HCIR Stage 1: Cue Layer Extraction Prompt

| Item | Content |
| --- | --- |
| Role | You are a personality psychologist specializing in the Big Five model and structured behavioral cue extraction. |
| Task | Below are five spoken responses from a participant who viewed a picture with numbered characters and answered the following prompts: (1) which character is most like you in a work context and why; (2) which character is most like you in a family context and why; (3) which character is most like you in a friendship context and why; (4) which character do you like most and why; (5) which character do you like least and why. |
| Instruction | For each of the five responses, classify the following information: (1) Prompt function — whether the response is primarily self-projection or evaluation; (2) Source type — whether the response draws on self-behavior, self-value, moral norm, or other; (3) Self-relevance — whether the response is directly self-referential, idealized/aspirational, low-self (e.g., describing others without self-insertion), or other; (4) Domain coverage — which of the Big Five domains (Extraversion, Agreeableness, Conscientiousness, Neuroticism, Openness) are relevant to this response; (5) Quality indicators — clarity, coherence, detail level, and whether the response engages meaningfully with the prompt. |
| Output Format | Provide a structured summary per prompt in a table format. |

Table A4. HCIR Stage 2: Trait-Specific Cue Extraction Prompt

| Item | Content |
| --- | --- |
| Role | You are a personality psychologist specializing in the Big Five model and trait-specific behavioral cue identification. |
| Task | For each of the Big Five domains below, identify specific cues from the participant's five responses that are relevant to that trait. |
| Instruction | For each cue, provide: (1) the cue text (a direct quote or close paraphrase from the response), (2) the prompt it came from (work, family, friends, liked, or disliked), and (3) a brief justification for why this cue is relevant to the trait. Be conservative — only extract cues that have a clear theoretical or empirical link to the trait. |
| Trait Definitions | Extraversion: sociability, assertiveness, energy, positive emotionality, social visibility. Agreeableness: warmth, trust, cooperation, prosocial stance, conflict avoidance. Conscientiousness: organization, responsibility, reliability, goal-directedness, task persistence. Neuroticism: anxiety, emotional instability, vulnerability, negative emotionality, reactivity. Openness: curiosity, imagination, aesthetic sensitivity, intellectual exploration, unconventional thinking. |
| Output Format | Provide a bulleted list per trait, with cue text, source prompt, and justification for each cue. |

Table A5. Inference Parameters for All LLM Calls

| Parameter | HAR Stage 1 | HAR Stage 2 | HCIR Stage 1 | HCIR Stage 2 |
| --- | --- | --- | --- | --- |
| Model | OpenAI GPT-5.4 / DeepSeek 3.2 | OpenAI GPT-5.4 / DeepSeek 3.2 | DeepSeek 3.2 / OpenAI GPT-5.4 | DeepSeek 3.2 / OpenAI GPT-5.4 |
| Temperature | 0.7 | 0.3 | 0.7 | 0.7 |
| Top-p | 0.9 | 0.95 | 0.9 | 0.9 |
| Max tokens | 512 | 256 | 512 | 512 |
| Response format | Structured text | Integer (1-9) | Structured text | Structured text |

Figure S1. Workflow diagram of the LLM-based pipelines (HAR and HCIR).

Note: The original prompts were administered in Chinese; the English versions shown here are translations for reproducibility.
